# Efficacy of a standardized tube weaning program in pediatric patients with feeding difficulties after successful repair of their esophageal atresia/tracheoesophageal fistula

**DOI:** 10.1007/s00431-020-03673-w

**Published:** 2020-05-15

**Authors:** Sabine Marinschek, Karoline Pahsini, Victor Aguiriano-Moser, Marion Russell, Barbara Plecko, Eva Z. Reininghaus, Holger Till, Marguerite Dunitz-Scheer

**Affiliations:** 1 Department of Psychiatry and Psychotherapeutic Medicine, Medical University of Graz, Auenbruggerplatz 31, 8010 Graz, Austria; 2 Department of Paediatrics and Adolescent Medicine, Division of General Pediatrics, Medical University of Graz, Auenbruggerplatz 34/2, 8010 Graz, Austria; 3 School of Pharmacy and Health Professions, Creighton University, 2412 Cuming sT #201, Omaha, NE 68131 USA; 4 Department of Paediatric and Adolescent Surgery, Medical University of Graz, Auenbruggerplatz 34, 8010 Graz, Austria

**Keywords:** Esophageal atresia, Tracheoesophageal fistula, Enteral nutrition, Tube weaning, Online coaching

## Abstract

Children born with esophageal atresia (EA) might suffer from significant oral feeding problems which could evolve into tube dependency. The primary aim of the study was to define the outcome of tube weaning in children after successful EA repair and to compare outcomes in children with short gap/TEF (tracheoesophageal fistula) and long-gap EA. Data of 64 children (28 with short-gap EA/TEF with primary anastomosis and 36 with long-gap EA with delayed surgical repair) who participated in a standardized tube weaning program based on the “Graz model of tube weaning” (in/outpatients in an intensive 3-week program, online coaching (Netcoaching) only, or a combined 2-week intensive onsite followed by online treatment “Eating School”) from 2009 to 2019 was evaluated. Sixty-one patients completed the program by transitioning to exclusive oral intake (95.3%). Three children (4.7%) were left partially weaned at the time of discharge. No significant differences could be found between short gap/TEF and long-gap EA group regarding outcomes.

*Conclusions*: The study’s findings support the efficacy of tube weaning based on the published “Graz model of tube weaning” for children born with EA/TEF and indicate the necessity of specialized tube weaning programs for these patients.

**Table:** 

**What is Known:** *• Children with esophageal atresia/tracheoesophageal fistula often suffer from feeding problems and tube dependency.* *• Different tube weaning programs and outcomes have been published, but not specifically for children with EA.*	
**What is New:** *• Evaluation of a large sample of children referred for tube weaning after EA repair.* *• Most children with EA can be weaned off their feeding tubes successfully after attending a specialized tube weaning program.*

**What is Known:**

*• Children with esophageal atresia/tracheoesophageal fistula often suffer from feeding problems and tube dependency.*

*• Different tube weaning programs and outcomes have been published, but not specifically for children with EA.*

**What is New:**

*• Evaluation of a large sample of children referred for tube weaning after EA repair.*

*• Most children with EA can be weaned off their feeding tubes successfully after attending a specialized tube weaning program.*

## Introduction

Esophageal atresia (EA) represents a congenital defect caused by abnormal embryological maturation of the upper gut/foregut leading to a blind-ending pouch and missing connection between the esophagus and the stomach. The spectrum of EA classification distinguishes anatomically between short gap/tracheoesophageal fistula (TEF) and long-gap EA. The prevalence of EA/TEF ranges between 1.27 and 4.55 per 10,000 births. In children with isolated EA/TEF, the survival rate is high (approaching 100%) [1]. Children with comorbidities such as prematurity, low birth weight, or additional congenital anomalies show higher mortality rates [1]. While short gap/TEF is usually reconstructed primarily by anastomosing both ends, special techniques are used to establish intestinal continuity in children with long-gap EA with no fistula [2]. Elongation procedures, such as repetitive bouginage, Kimura, or traction sutures, as proposed by Dr. Foker, aim to elongate both esophageal ends until a delayed primary anastomosis is feasible [3]. Esophageal replacement techniques are advocated in ultra-long-gap EA to allow for an oral passage via a conduit like gastric pull-up or colonic and small bowel interposition [3]. Postoperative complications remain a major challenge in children with EA despite excellent surgical and neonatal management. Dilatations due to anastomotic strictures, revisional surgery due to leakage, or, in rare cases, fistula relapse may prolong parenteral or gastrostomy tube feeding [1]. The use of feeding tubes is mandatory to ensure nutrition and growth of these patients until oral nutrition is possible [4, 5]. A gastrostomy is often placed in the first days of life [1], particularly in cases of long-gap EA. As a result, some patients may miss the chance to acquire a natural swallowing mechanism [6–8]. Furthermore, motility problems are quite frequent in children with esophageal atresia [6].

The use and indication of enteral nutrition by feeding tubes in pediatric patients has increased greatly in the last 30 years [4, 9]. Unfortunately, side effects of tube feeding occur frequently and significantly diminish the overall quality of life [10], as the material itself can irritate the gastrointestinal mucosa leading to irritation, regurgitation, and reflux [4, 5]. Chronic inflammation of the mucosa, tube dislocations, nausea, reflux, gagging and retching, as well as oral aversion and food refusal are other frequently reported side effects [11]. In consequence, children requiring long-term tube feeding often do not develop the normal developmental abilities of sucking, biting, chewing, and swallowing solid food. These children may develop tube dependency, defined as an “unintended result of long-term tube feeding in infants and young children […]. It prevents infants from making the transition from tube to oral feeding despite the absence of any medical indication for continuation of enteral feeding” and may result in oral and tactile aversion, food refusal, recurrent retching/gagging/vomiting, and failure to thrive [4]. Families of children with these issues suffer great stress and isolation [4, 8, 12, 23]. A variety of tube weaning methods have been described and discussed [13–18] in the literature. Because a dearth of specific information about tube weaning in patients with esophageal atresia exists in recent literature, the authors decided to take a closer look at this population.

With a success rate (defined at discharge as full und sufficient oral intake 35 days after last tube feed/tube removal, with the child in good general state and stable weight condition) of over 90%, the “Graz model of tube weaning” is a carefully designed tube weaning method characterized by child led and autonomy supporting techniques [4, 16, 19, 20]. The “Graz model of tube weaning” is based on two main principles:

1. Enable the child to feel hunger
2. Enable the child’s autonomy and encourage oral intake

### Ad 1: enable the child to feel hunger

Tube feeds are reduced individually under daily medical supervision, based on age, growth, state, medical condition, and possible pre-existing oral skills of the child. Weight and intake are evaluated on a daily basis by the medical professionals. Generally, a quick reduction of tube feeds is preferred in order to avoid habituation effects and long-term failure to thrive.

### Ad 2: enable the child’s autonomy and encourage oral intake

Since tube dependent children often develop severe avoidance and refusal patterns, the use of their hands as natural feeding instruments as well as the technical and practical aspects of food intake must be developed from scratch. An important part of the weaning process is the “play picnic,” where different kinds of food are served in a playful manner, with or without little plates, and children are allowed to interact with food (touch, smell, throw, lick, etc.) without any expectation of intake or restriction or interference by adults. The motto is “as little help as possible but as much as necessary.”

The “Graz model of tube weaning” offers intensive treatment, including multiple daily interactions with a therapeutic team made up of pediatricians, psychologists, and feeding therapists. Furthermore, children receive therapy in individual and group settings, and all families get psychological/psychotherapeutic support. In the online tele-medical program (Netcoaching), a pediatrician, clinical psychologist, and feeding therapist support each family on a daily basis with at least one asynchronous contact per 24-h period. The ultimate goal of the “Graz mode of tube weaning” is the stable and sustainable establishment of self-regulated oral intake [21].

The main aim of this study was to evaluate the outcome of the “Graz model of tube weaning” for children with EA. The second aim was to compare success rates between children in the short gap/TEF group and the long-gap EA group after completion of the tube weaning program.

## Material and methods

Inclusion criteria for the study were patients born with EA who had undergone successful surgical reconstruction and who participated in a tube weaning program based on the “Graz model of tube weaning” between 2009 and 2019. Treatment options were [1] in- or outpatient treatment at the University Hospital for Children and Adolescents, Department of General Pediatrics in Graz, Austria, [2] combined onsite and online treatment at NoTube Interdisciplinary Therapy Center for Eating and Feeding Disorders, Graz, Austria, or [3] online treatment only (Netcoaching by NoTube non-profit LLC, Graz, Austria). Data was obtained from local medical records. The research ethics board of Medical University of Graz provided written approval for the study (EK-31-072 ex 18/19).

### Objectives

The primary aim of the study was to evaluate the success rate of “Graz model” tube weaning programs in children with EA. For this aim of the study, outcome variables were defined as the success rate after completion of the tube weaning program (35 days after the very last tube feed/tube removal, sufficient oral intake calculated by a pediatrician, stable weight condition, good general state with a discharge letter to the pediatrician stating expected growth, and when to contact the treatment team again):

- Outcome 1, exclusive oral nutrition, no more tube feeding
- Outcome 2, oral nutrition combined with supplement by tube

To address the second aim of the study, comparing success rates between children with short gap/TEF and children with long-gap EA, participants were divided into two subgroups. Group 1 consisted of children with “short gap/TEF” with primary anastomosis (*n* = 28, 43.75%) surgery during the first days of life. Group 2 consisted of children with “long-gap EA,” with delayed surgical repair (*n* = 36, 56.25%).

### Data analysis

Data was collected utilizing the RDA System at the Medical University of Graz (administration, Medical Informatics, Statistics, and Documentation) and analyzed via SPSS V25.0 (SPSS, Chicago, IL, USA). Descriptive statistics, Mann-Whitney *U* test, Wilcoxon test, Kruskal-Wallis test, and *t* test were used for group differences. Contingency tables for categorical and non-parametric data (Chi-squared tests, Fisher’s exact tests) were calculated. A *p* value of < 0.05 was considered statistically significant.

#### Description of the sample

Sixty-four patients (34 males, 53.1% and 30 female, 46.9%) participated in the study. All participants underwent a tube weaning program based on the “Graz model” between 2009 and 2019. Children in group short gap/TEF were significantly younger (*MD* = 1.04 years) at admission than those in long-gap EA (*MD* = 1.39 years).

Of the 64 children, 32 (50.0%) participated in the online only Netcoaching program, 23 (35.9%) in the 2-week, intensive, onsite Eating School program, 5 (7.8%) in the inpatient program, and 4 (6.3%) in the outpatient treatment program. Patients were referred from 16 different countries, with the greatest number coming from Germany (*n* = 23; 35.9%) and Austria (*n* = 9; 14.1%).

Table 1 shows demographic and clinical parameters of the sample.

**Table 1:** Demographic and clinical parameters

Parameters	Short gap/TEF (*n* = 28) M (SD)	Long gap EA (*n* = 36) M (SD)	Statistics	*p* value
Sex (%)				
Male	13 (46.4)	21 (58.3)	*X*^*2*^(1) = 0.896	0.450
Female	15 (53.6)	15 (41.7)		
Age (years) (MD)	1.04	1.39	*U* = 346.5	0.030*
Treatment (%)				
Netcoaching	12 (42.9)	20 (55.6)		
Eating School	12 (42.9)	11 (30.5)	*Fisher’s* = 2.29	0.557
Inpatient program	3 (10.7)	2 (5.6)		
Outpatient program	1 (3.6)	3 (8.3)		
COF—Top 5 (%)				
Germany	10 (35.7)	13 (36.1)		
Austria	5 (17.9)	4 (11.1)		
France	3 (10.7)	4 (11.1)	*Fisher’s* = 12.14	0.770
USA	1 (3.6)	3 (8.3)		
Australia	1 (3.6)	3 (8.3)		
Others^#^	8 (28.5)	9 (25.1)		
Delivery (%)				
Caesarian section	15 (53.6)	28 (77.8)		
Spontaneous vaginal delivery	10 (35.7)	6 (16.7)	*Fisher’s* = 5.595	0.170
Forceps delivery	2 (7.1)	1 (2.75)		
Missing data	1 (3.6)	1 (2.75)		
GA (weeks) (M(SD))	35.39 (3.67)	34.39 (3.88)	*t*(62) = 1.052	0.297
Full term (%)	9 (32.1)	9 (25.0)		
Preterm (%)	18 (64.3)	23 (63.9)	*X*^2^(2)=1.432	0.489
Extremely preterm (%)	1 (3.6)	4 (11.1)		
Feeding tube route (%)				
PEG	13 (46.4)	18 (50.0)	*X*^2^(2)=1.722	0.415
NG	10 (35.7)	8 (22.2)		
Jejunal	5 (17.9)	10 (27.8)		
Nutrition (%)				
Tube fed since birth	26 (92.2)	31 (86.1)		
Ate orally (> 1 week)	2 (7.1)	2 (5.6)	*Fisher’s* = 2.209	0.401
Missing data	0	3 (8.3)		
Formula (tube) (%)				
Normocaloric tube formula	9 (32.1)	10 (27.8)		
(Fortified) baby milk	5 (17.8)	8 (22.2)		
High caloric tube formula	6 (21.5)	8 (22.2)		
(Fortified) breast milk	3 (10.7)	2 (5.6)		
Pureed food	3 (10.7)	3 (8.4)	*Fisher’s* = 5.104	0.944
Peptide-based formula	1 (3.6)	3 (8.4)		
Amino-based infant formula	1 (3.6)	1 (2.8)		
Ketogenic diet	0	1 (2.8)		

Results from Chi-squared tests and Kruskal-Wallis test (*X*^*2*^(df)); Mann-Whitney *U* tests and Wilcoxon test (*U*); *t* test (*t*(df)). Fisher’s (*Fisher’s exact test*). *p < 0.05. COF, country of referral. ^#^includes all other countries: Switzerland, Great Britain, Hungary, Ireland, Italy, Luxembourg, Martinique, Netherlands, Romania, Serbia, South Africa

No significant differences for the two groups were found regarding demographic and clinical parameters except for the age at admission (Table 1).

#### Prior surgical treatment of EA/TEF

In many cases, a primary anastomosis of the esophagus was possible within the first few days of life (*n* = 28, 43.8% group short gap/TEF), whereas in the group long-gap EA, 36 children (56.2%) had to wait for two to 18 months to undergo surgical repair. Two children with long-gap EA underwent gastric transposition, two colonic interposition, and one jejunal interposition.

#### Peri-/postoperative complications/treatment

An overview on peri- and postoperative complications and treatment can be found in Table 2.

**Table 2:** Peri-/postoperative complications/treatment

Peri-/postoperative complications/treatment	Short gap/TEF (*n* = 28) frequencies (%)	Long-gap EA (*n* = 36) frequencies (%)
Multiple dilatations	6 (21.4)	17 (42.7)
Tracheostomy*	6 (21.4)	0
Fundoplication (Nissen, Thal)	4 (14.3)	8 (22.2) **
Fistular relapses with surgical ligation	4 (14.3)	2 (5.6)
Esophageal stent implantation	2 (7.1)	0
Dislocation of the stent	1 (3.6)	0
Anastomosis insufficiency	1 (3.6)	0
Dumping syndrome	1 (3.6)	2 (5.6)
Pylorospasm/delayed gastric emptying	0	1 (2.8)
Esophageal perforation	0	1 (2.8)
Klebsiella infection during EA surgery	0	1 (2.8)
Bronchial ligation, partial lung resection	0	1 (2.8)
Anastomosis perforation	0	1 (2.8)
Anastomosis surgery unsuccessful	0	1 (2.8)
Gastric pull-up failed	0	1 (2.8)

Frequencies only; EA, esophageal atresia; TEF, tracheoesophageal fistula; * tracheostomy placed due to paralysis of the vocal cord after EA repair (*n* = 4), laryngeal stenosis (*n* = 1), laryngeal cleft (*n* = 1); ** re-fundoplication necessary (*n* = 2), re-re fundoplication necessary (*n* = 1)

#### Comorbidities

Many children suffered from additional medical problems alongside the esophageal atresia (multimorbidity). Further information is listed in Table 3.

**Table 3:** Comorbidities

Comorbidities	Short gap/TEF (*n* = 28) frequencies (%)	Long-gap EA (*n* = 36) frequencies (%)
Prematurity	19 (67.9)	27 (75.0)
GERD	6 (21.4)	6 (16.7)
Duodenal stenosis/atresia	1 (3.6)	5 (13.9)
VSD, ASD	3 (10.7)	5 (13.9)
IUGR	4 (14.3)	5 (13.9)
Paralysis of vocal chord	4 (14.3)	0
VACTERL association	3 (10.7)	3 (8.4)
Tracheomalacia	2 (7.1)	3 (8.4)
Lung hypoplasia	1 (3.6)	3 (8.4)
Choanal atresia	3 (10.7)	0
Hearing loss	2 (7.1)	3 (8.4)
Down syndrome	1 (3.6)	2 (5.6)
Hydrocephalus	1 (3.6)	2 (5.6)
Cleft palate	2 (7.1)	1 (2.8)
Double outlet right ventricle	2 (7.1)	1 (2.8)
Anal atresia	2 (7.1)	0
CHARGE syndrome	2 (7.1)	0
Mandibular dysostosis	1 (3.6)	1 (2.8)
Pulmonary stenosis	1 (3.6)	0
Laryngeal cleft	1 (3.6)	0
Laryngotracheal stenosis	1 (3.6)	0
Malformation syndrome	1 (3.6)	0
NEC	1 (3.6)	0
Omphalocele	1 (3.6)	0
Subglottic stenosis	1 (3.6)	0
Cerebral palsy	1 (3.6)	0
Chiari malformation	1 (3.6)	0
Coarctation of the aorta	0	1 (2.8)
Blindness	0	1 (2.8)
Encephalopathy	0	1 (2.8)
Lennox-Gastaut syndrome	0	1 (2.8)
Tetralogy of Fallot	0	1 (2.8)

Frequencies only; EA, esophageal atresia; TEF, tracheoesophageal fistula

## Results

### Tube weaning outcomes

**Fig. 1:**
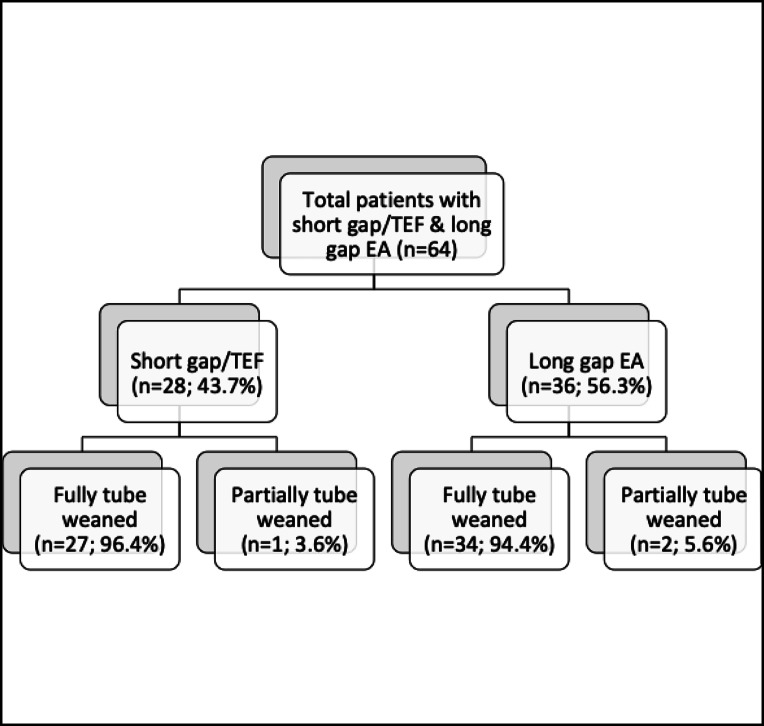
Flow chart of the main outcome variables

A total of 61 patients (95.3%), 27 with short gap/TEF and 34 with long-gap EA, completed the tube weaning program having successfully transitioned to exclusive oral nutrition (outcome 1) (Fig. 1). Three children (4.7%) remained on partial tube supplements (mostly at night) at the time of discharge (outcome 2) (Fig. 1). No significant differences were shown between the short gap/TEF and long-gap EA groups regarding outcome (Table 4).

**Table 4:** Tube weaning Outcomes

Tube weaning outcomes (%)	Short gap/TEF (n = 28)	Long gap EA (n = 36)	Statistics	*p* value
Outcome 1	27 (96.4)	34 (94.4)		
Outcome 2	1 (3.6)	2 (5.9)	*X*^2^(1) = 0.139	0.709
Total Outcome 1^###^	61 (95.3)		–	–

Results from Chi-squared tests (*X*^*2*^(df)). Outcome 1, fully tube weaned. Outcome 2, partially tube weaned. ^###^ Overall tube weaning outcome (*N* = 64)

The reasons for the three children staying on partial tube feeds were as followed:

1. One child was discharged on night feeds due to the parents’ decision to complete the final weaning at home. The child was on exclusive oral nutrition 2 months later.
2. Partial weaning was considered the best possible option for one child who was not able to sustain themselves fully by oral intake at the time of discharge—completion of the full weaning process was planned for after closure of his tracheostomy.
3. For one child, partial weaning was considered the only possible option, as the patient had multiple comorbidities that precluded exclusive oral nutrition.

On average, patients lost weight during the weaning process (MD − 2.42% of their starting weight compared with weight at discharge) but grew in length (MD + 1.52% from treatment start to discharge). No significant differences between the short gap/TEF and long-gap EA and weight development in % could be detected (see Table 5).

**Table 5:** Biometric data

Biometric data	MD	Min.*	Max.**	Statistics	*p* value
Weight development (in %)					
Short gap/TEF (*n* = 27)	0	− 12.90	71.15		
Long-gap EA (n = 36)	− 3.33	− 13.28	48.30	*U* = 376.00	0.127
Total	− 2.42	− 13.28	71.15		
Height development (in %)					
Short gap/TEF (n = 27)	**3.17**	0	23.33		
Long gap EA (*n* = 35)	**1.22**	0	10.59	*U* = 306.00	0*.*016*
Total	1.52	0	23.33	n.a	n.a

Results from Mann-Whitney *U* tests (*U*). Statistically significant effects are marked bold. *p < 0.05. * Child with greatest weight loss from treatment start to discharge; **child with greatest weight gain from treatment start to discharge

Median duration of tube weaning treatment was 62.5 days. No significant difference in duration of treatment between short gap/TEF and long-gap EA group were shown. Mean duration for the Netcoaching (online treatment only) was 96.5 days (*SD* = 81.33) whereas Eating School (onsite followed by online treatment) lasted on average for 184.7 days (*SD* = 179.14). Patients who participated in the combined onsite and online treatment needed significantly longer treatment than those participating in the online only program.

## Discussion

This single-center study aimed to evaluate outcomes of children with EA who underwent a tube weaning program based on the “Graz model” and to compare the outcomes of tube weaning between children with short gap/TEF and long-gap EA. The study provided first data on a large international sample of patients with tube dependency after surgical correction of EA in different centers. The study demonstrated that more than 95% of all children could be weaned successfully and needed no tube feeds after completion of the tube weaning program, with three children requiring partial tube feeds.

Previous studies have shown that many children with EA, especially those with long-gap EA, suffer from feeding difficulties [7, 8, 22] and that some of these children are discharged on home ENS [8]. Many children with EA succeed in the transition to oral nutrition on their own; however, a large proportion—especially among those with comorbidities [8]—does not succeed in transitioning to oral intake despite implementation of various supportive measures. These children develop feeding tube dependency that requires specialized treatment. In fact, over 40% of all children referred to tube weaning programs were born with short gap/TEF receiving primary anastomosis. The initial assumption of increased incidence of eating disturbances and severe limitations in eating development in the group of the long gap EA patients could not be confirmed in this study. Superficially, this finding might seem surprising, but this should be critically interpreted since other influencing variables, such as sensory perception issues, degree of delayed general development, and relationship and family patterns, may also have a crucial impact on these findings.

This study showed the promising result of over 95% of all children with EA being successfully weaned off their feeding tubes completely after participating in tube weaning program based on the “Graz model.” This finding was independent of age, gender, type of feeding tube, or birth variables. Furthermore, little weight loss (2.42%) occurred during the weaning process.

The major strength of this study is its large international sample size, which is the most representative of this population published to date with regard to tube weaning.

A weakness of the study was that it did not describe individual weight and height data. Authors chose not to include this data based on the opinion that an overview about weight development would be more reliable. Furthermore, the study did not include long-term outcomes, e.g., months or years after discharge, with respect to feeding, weight, and growth development. While long-term outcomes of the “Graz Model of tube weaning” have been recently published [24], a specific analysis for those children born with EA may be beneficial for the future.

In conclusion, the present findings of the study support the efficacy of tube weaning based on the “Graz model of tube weaning” for children with both short gap/TEF and long-gap EA. As soon as a child has recovered from surgical repair and is medically stable and cleared for oral intake, tube weaning should be undertaken. Early oralization and a tube weaning program supervised by specialized medical professionals are of utmost importance for children with EA.

## Abbreviation

ASD: Atrial septal defect
COF: Country of referral
EA: Esophageal atresia
GA: Gestational age
IUGR: Intrauterine growth retardation
M: Mean
MD: Median
NG tube: Nasogastric tube
PEG: Percutaneous endoscopic gastrostomy
SD: Standard deviation
TEF: Tracheoesophageal fistula
VSD: Ventricular septal defect
